# Linear Low-Dose Extrapolation for Non-Cancer Responses: Burke et al. Respond

**DOI:** 10.1289/ehp.0800329R

**Published:** 2009-04

**Authors:** Thomas A. Burke, Francesca Dominici, Mary Fox, Ronald H. White, Ila Cote, Dale B. Hattis, Jonathan M. Samet, Paul D. White, Lauren Zeise

**Affiliations:** Johns Hopkins Bloomberg School of Public Health, Baltimore, Maryland, E-mail: rwhite@jhsph.edu; U.S. Environmental Protection Agency, Research Triangle Park, North Carolina; Clark University, Worcester, Massachusetts; University of Southern California, Los Angeles, California; U.S. Environmental Protection Agency, Washington, DC; California Environmental Protection Agency, Sacramento, California

In his letter, Rhomberg raises several issues concerning recommendations in our report of the workshop “Issues and Approaches to Low Dose–Response Extrapolation for Environmental Health Risk Assessment” (White et al. 2009). One recommendation of the workshop was to set aside the generally held presumption that dose–response functions should follow a threshold model when extrapolating from higher dose studies of non-carcinogenic responses to lower dose levels typical for environmental exposures to chemicals. Workshop participants generally concluded that the selection of population-level low-dose extrapolation models should be informed by population factors such as inter individual variability in susceptibility and coexposures, as well as by categorization of mechanisms of toxicity. As indicated in the meeting report (White et al. 2009), most workshop participants preferred a linear, no-threshold approach to low-dose extrapolation modeling, combined with modeled estimates of the low range of observed data, for noncancer, as well as cancer, outcomes in the absence of convincing evidence to indicate that an alternative model is more appropriate. We recognize that this recommendation represents a departure from current generally accepted practice.

On a nonsubstantive point, Rhomberg’s comment that we did not include additional information regarding “fuller discussions” at the workshop on this and other issues reflects the constraints imposed by *EHP*’s article length limits and changes made to accommodate reviewer comments encouraging emphasis on workshop findings and recommendations rather than on workshop discussions.

We disagree with Rhomberg’s assertion that the finding of a linear, no-threshold exposure–response relationship in many epidemiologic studies of the effect of environmental pollutants, such as particulate matter and ozone air pollution, can be attributed entirely to a small range of exposures and measurement error. Although these factors need to be considered in evaluating epidemiologic study results, modeling techniques such as non parametric smoothing methods have demonstrated the capacity to identify potential threshold relationships even in the context of relatively extreme measurement error (Cakmak et al. 1999; Schwartz and Zanobetti 2000).

As we noted in our meeting report (White et al. 2009), for the limited number of chemicals and agents for which robust low-dose response data exists (e.g., epidemiologic studies of large populations with exposures to particulate matter and ozone air pollution extending from relatively high to low ambient levels), thresholds have not been observed for non cancer or cancer outcomes [U.S. Environmental Protection Agency (EPA) 2006a, 2006b]. Additionally, for some of the exposures considered, the mechanisms of action thought to underlie the observed effects have been characterized by some as threshold mechanisms (e.g., the disruption of the homeostatic conditions for reactive oxygen species). In such cases, interindividual variability, background disease processes, and coexposures may explain the observed linearity.

Although we acknowledge that there are differences in the intrinsic biological processes involved in generating cancer and non cancer outcomes, we disagree with Rhomberg’s assertion that heterogeneity in intrinsic population susceptibility and additivity to background disease processes result in simply “broadening” the dose–response relationship (which we presume means making the dose–response curve shallower). The underlying concept that additivity to background disease processes and variability in population susceptibility results in a linearization of the dose–response function for populations exposed to environmentally relevant levels was originally discussed in the context of cancer outcomes [Crump et al. 1976; Lutz 1990; National Research Council (NRC) 2005], and the suggestion that this same concept applies to noncancer outcomes is not novel (Clewell and Crump 2005; Crawford and Wilson 1996). Similarly, the importance of considering interindividual variability in assessing uncertainty associated with chemical risk assessments of noncancer effects has been recognized (Hattis and Silver 1994). The significance of these factors in the selection of dose–response models for use in environmental health risk assessment was also highlighted in a recent NRC report (NRC 2008).

Regarding the assumption of additivity to background disease on low-dose extrapolation, in our meeting report (White et al. 2009) we noted the importance of assessing, to the extent possible, whether the mode or mechanism of action of the key events involved are consistent. However, current knowledge of these detailed biologic processes is still quite limited for most chemicals and pollutants, and as noted by Hoel (1997)

[L]ow-dose linearity is speculative and it is a reasonable assumption for public health purposes in those instances where there is no scientific evidence to the contrary.

We recognize that uncertainty increases as the dose–response extrapolation extends farther below observed data. The findings regarding exposure–response relationships from large-scale epidemiologic studies of environmental pollutants suggest that when considering population-level dose–response factors, interindividual variability, additivity to background disease processes, coexposures, and mechanisms of action, warrant careful consideration. As a consequence, we continue to recommend that the approach proposed in our meeting report (White et al. 2009) is appropriate and necessary.
